# Supplementary material for: Incommensurate spin correlations in highly oxidized cobaltates La2−xSrxCoO4
Source: Sci Rep. 2016 Apr 27;6:25117. doi: 10.1038/srep25117 (PMC4846828; doi:10.1038/srep25117)
Supplement: Supplementary Information [file srep25117-s1.pdf]

Supplementary Materials for

Incommensurate spin correlations in highly oxidized cobaltates $La_{2-x}Sr_xCoO_4$

Z. W. Li[1] , Y. Drees [1], C. Y. Kuo[1], H. Guo[1], A. Ricci[2], D. Lamago[3,4], O. Sobolev[5,6], U. Rütt 2 , O. Gutowski[2], T. W. Pi[7], A. Piovano[8], W. Schmidt[8,9], K. Mogare[1], Z. Hu[1] , L. H. Tjeng[1], and A. C. Komarek[1,*]

[1] Max-Planck-Institute for Chemical Physics of Solids, Nöthnitzer Str. 40, 01187 Dresden, Germany

[2] Deutsches Elektronen-Synchrotron DESY, Notkestr. 85, 22603 Hamburg, Germany

[3] Forschungszentrum Karlsruhe, Institut für Festkörperphysik, P.O.B. 3640, D-76021 Karlsruhe, Germany

[4] Laboratoire Léon Brillouin, CEA/CNRS,F-91191 Gif-sur Yvette Cedex, France

[5] Forschungsneutronenquelle Heinz Maier-Leibnitz (FRM-II), TU München, Lichtenbergstr. 1, D-85747 Garching, Germany

[6] Georg-August-Universität Göttingen, Institut für Physikalische Chemie, Tammannstrasse 6, D-37077 Göttingen, Germany

[7] National Synchrotron Radiation Research Center (NSRRC), Hsinchu 30077, Taiwan

[8] Institut Laue-Langewin (ILL), 71 avenue des Martyrs, F-38042 Grenoble Cedex 9, France

[9] Jülich Centre for Neutron Science JCNS, Forschungszentrum Jülich GmbH, Outstation at ILL, 71 avenue des Martyrs, F-38042 Grenoble Cedex 9, France

* Alexander.Komarek@cpfs.mpg.de

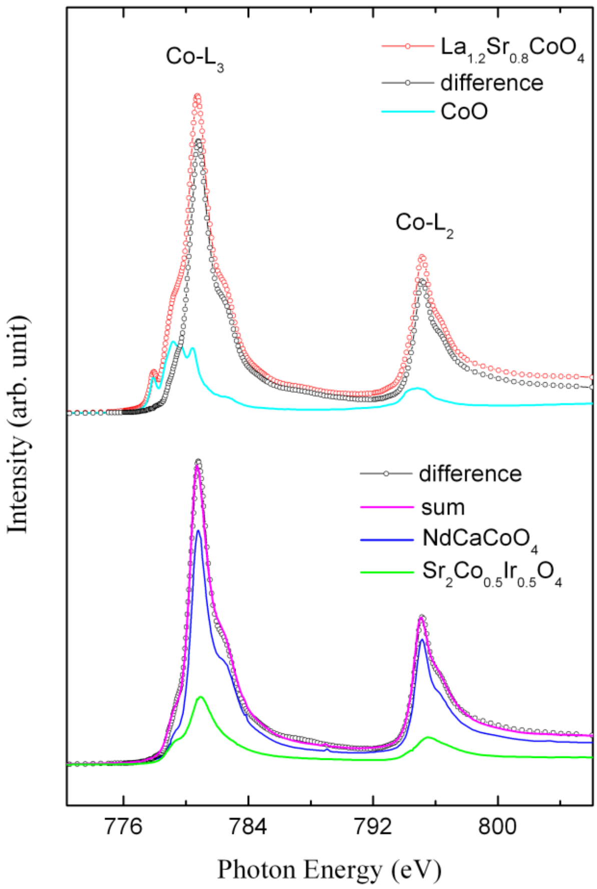

Fig. S1 The Co-$L_{2,3}$ XAS spectrum of $La_{1.2}Sr_{0.8}CoO_4$ is shown together with XAS spectra of CoO, $NdCaCoO_4$ and $Sr_2Co_{1/2}Ir_{1/2}O_4$ reference samples. The pre-peak at 778 eV can be attributed to the presence of a Co^{2+} species. The experimental Co-$L_{2,3}$ spectrum of $La_{1.2}Sr_{0.8}CoO_4$ can be decomposed into 77% Co^{3+} content, denoted as 'difference' (black circles) and 23% Co^{2+} contribution (cyan line). These measurements show that our $La_{1.2}Sr_{0.8}CoO_{4+\delta}$ sample has only a tiny oxygen deficiency (δ=-0.015±0.005). After subtraction of the Co^{2+} contribution (denoted as 'difference'), we were able to estimated the HS content for the Co^{3+} species. Therefore, we used $NdCaCoO_4$ and $Sr_2Co_{0.5}Ir_{0.5}O_4$ [S1,S2] as a Co^{3+} LS and Co^{3+} HS reference material, respectively. The lower part of this figure shows that the difference spectrum can be nicely reproduced by the weighted sum (magenta) of the Co^{3+} HS spectrum (green line) and the Co^{3+} LS spectrum (blue line). Thus, the presence of ~19% Co^{3+} ions in the HS state and ~58% Co^{3+} ions in the LS state is revealed by this modelling of the 'difference' spectrum (besides 23% Co^{2+} ions).

References

[S1] X. Ou and Hua Wu, Phys. Rev. B **89**, 035138 (2014)

[S2] unpulished results

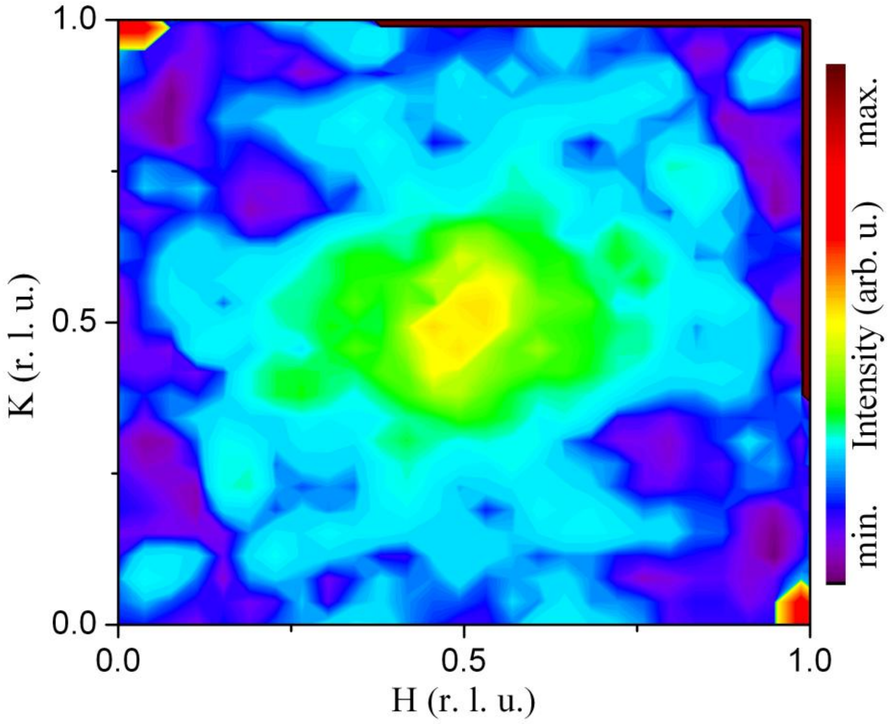

Fig. S2 Fourrier transformation of charge ordering pattern used for numerical simulations in Fig. 6 (a). The expected intensity for charge correlations is shown in a logarithmic colour-contour plot. Since only the modulations of the Co-oxygen bond distances can be seen with neutrons and not the charges itself, the values are arbitrary.

Supplementary Video Legends

Supplementary Video #1: **Low energy magnetic excitations**

The movie shows a simulation of spin excitations at ~1.3 meV within our nano phase separation model for $La_{1.3}Sr_{0.7}CoO_4$.

Supplementary Video #2: **Higher energy magnetic excitations**

The movie shows a simulation of spin excitations at ~8.4 meV within our nano phase separation model for $La_{1.3}Sr_{0.7}CoO_4$.
