# Supplementary figures and images for: Incommensurate spin correlations in highly oxidized cobaltates La2−xSrxCoO4
Source: Sci Rep. 2016 Apr 27;6:25117. doi: 10.1038/srep25117 (PMC4846828; doi:10.1038/srep25117)

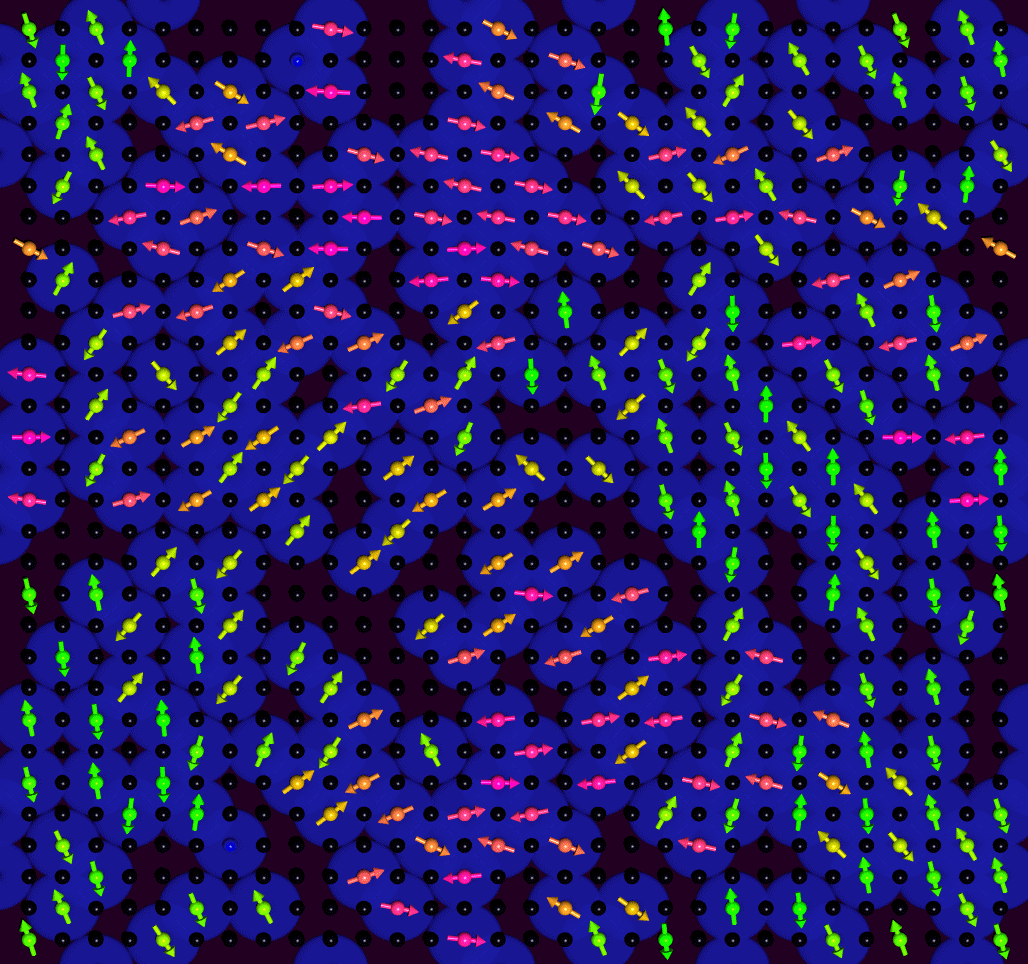

Supplement: Supplementary Video S1 [file srep25117-s2.gif]

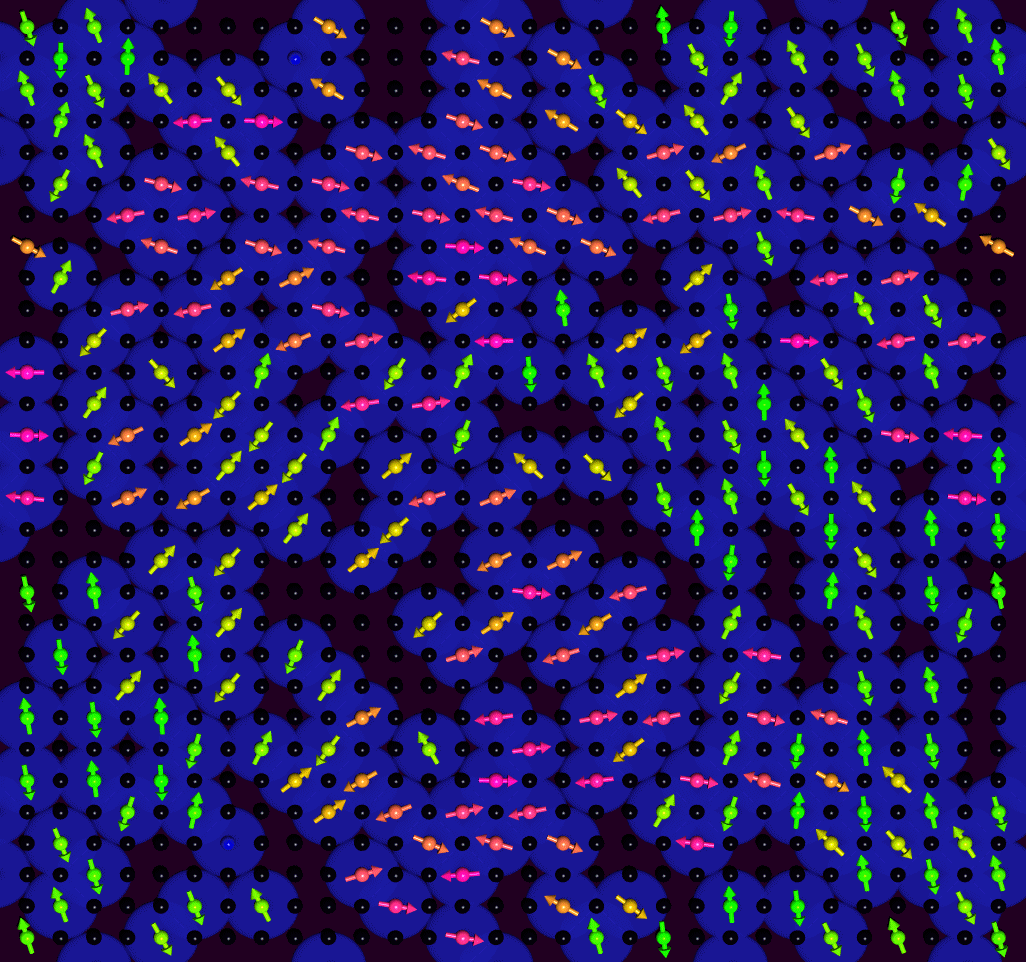

Supplement: Supplementary Video S2 [file srep25117-s3.gif]
